# Structural insights and biomedical potential of biosynthesized silver nanoparticles: antibacterial activity, anti-biofilm and cancer cell inhibition

**DOI:** 10.7717/peerj.19608

**Published:** 2025-07-01

**Authors:** Ikram Jemel, Najeh Krayem, Hajer Jlidi, Abir Ben Bacha, Mona Alonazi, Raihane Charguia, Areej Ali Alzahrani, Sami Aifa, Sami Mnif

**Affiliations:** 1 Laboratory of Molecular and Cellular Screening Processes, Centre of Biotechnology of Sfax, Sfax, Tunisia; 2 Laboratory of Biochemistry and Enzymatic Engineering of Lipases, ENIS, University of Sfax, Sfax, Tunisia; 3 Department of Biochemistry, College of Science, King Saud University, Riyadh, Saudi Arabia; 4 Department of Physics, College of Science, Qassim University, Buraydah, Saudi Arabia

**Keywords:** Plant extracts, Antibacterial activity, Anti-biofilm, Cancer cell inhibition, Nanotechnology, Silver nanoparticles

## Abstract

**Background:**

The increasing threat of antimicrobial resistance and cancer has driven the search for new therapeutic agents, with plant-based biosynthesis of nanoparticles emerging as a promising approach. Silver nanoparticles (AgNPs) synthesized from plant extracts have gained attention for their potential biomedical applications.

**Objective:**

This study aimed to synthesize, characterize, and evaluate the antimicrobial, anti-biofilm, and anticancer properties of AgNPs derived from *Teucrium polium* (Tp), *Teucrium marum* (Tm), and *Punica granatum* (Pg).

**Methods:**

AgNPs were synthesized using plant extracts and characterized by X-ray diffraction (XRD), dynamic light scattering (DLS), UV spectroscopy, and Fourier-transform infrared spectroscopy (FTIR). Antibacterial activity was assessed against Gram-negative and Gram-positive bacteria. The anti-biofilm efficacy was tested against *Staphylococcus aureus* and *Pseudomonas aeruginosa*. Cytotoxicity was evaluated on MCF-7 breast cancer cells.

**Results:**

XRD confirmed the face-centered cubic (fcc) crystal structure of AgNPs, with peaks at (111), (200), (220), and (311) planes. Crystallite sizes were smaller than their hydrodynamic diameters, suggesting surface modifications affecting DLS measurements. DLS analysis indicated monodisperse size distributions for Tp-AgNPs and Tm-AgNPs, while Pg-AgNPs showed a broader range. The biosynthesized silver AgNPs from different plants exhibit unique physicochemical properties, as evidenced by their distinct UV-vis absorption spectra, and show potential for optical and optoelectronic applications. FTIR spectroscopy analysis confirmed the presence of specific functional groups on the surface of biosynthesized AgNPs, indicating the role of plant extracts as reducing and capping agents, and revealed variations in binding sites and molecular interactions. Among the synthesized nanoparticles, Tp-AgNPs exhibited the highest antibacterial efficacy, particularly against *Staphylococcus epidermis* and *Pseudomonas aeruginosa*, though slightly less potent than chloramphenicol. Tp-AgNPs also showed the strongest inhibition of biofilm formation, followed by Tm-AgNPs, with Pg-AgNPs being the least effective. Cytotoxicity assays demonstrated that Tm-AgNPs had the highest anticancer activity against MCF-7 cells, with Tp-AgNPs exhibiting comparable effects.

**Conclusion:**

The findings underscored the potent antimicrobial, anti-biofilm, and anticancer properties of Tp-AgNPs and Tm-AgNPs, making them promising candidates for biomedical applications. Further studies are needed to assess their clinical safety and therapeutic potential.

## Introduction

Nanoparticles (NPs), defined as particles with dimensions smaller than 100 nm, exhibit unique physicochemical properties due to their high surface energy, large surface area-to-volume ratio, and structural stability (Mohanta et al., 2020). These characteristics make them excellent drug carriers by enhancing the solubility and bioavailability of poorly water-soluble compounds. Additionally, NPs enable targeted drug delivery through passive accumulation in tumor tissues *via* the enhanced permeability and retention effect, or through active targeting by surface functionalization with ligands that recognize specific cellular receptors (Altaf et al., 2021). Upon binding to cell surface receptors, nanocarriers are internalized into cancer cells *via* receptor-mediated endocytosis, enhancing intracellular drug accumulation. The efficiency of this delivery process is closely influenced by the structural and compositional features of the nanoparticles (Altaf et al., 2021). Various organic nanocarriers, including polymeric nanoparticles dendrimers, and lipid-based systems like liposomes, have been extensively explored due to their biocompatibility and controlled drug release properties. These carriers enhance biocompatibility and minimize toxicity risks in biomedical applications (Bhardwaj & Kaushik, 2017). On the other hand, inorganic nanomaterials, including silver, gold, platinum, iron, titanium, and zinc, are widely preferred for various biomedical applications, such as diagnostics and photothermal therapy, due to their unique physical and chemical properties (Bhardwaj & Kaushik, 2017). Silver has long been recognized for its potent antimicrobial properties against a broad spectrum of pathogens and has been used medicinally since ancient times. Today, silver nanoparticles (AgNPs) are being extensively studied for biomedical applications, including their incorporation into topical ointments, medical devices, and consumer products (Hashemi, Tasharrofi & Saber, 2020; Mohanta et al., 2020; Almayouf et al., 2024). While chemical synthesis enables rapid production of AgNPs, it often involves toxic reagents, harmful solvents, reducing agents, and expensive metal salts. Similarly, physical methods demand costly equipment and high energy, pressure, and temperature, limiting their scalability and environmental sustainability (Mohanta et al., 2020; Al-Dbass et al., 2022). To mitigate toxicity, reduce reliance on harmful reagents, and improve eco-friendliness, biological approaches utilizing microorganisms and plants are increasingly being explored for synthesizing metal nanoparticles, particularly for applications in products that contact human skin. These methods leverage natural reducing agents and biomolecules present in biological systems to facilitate nanoparticle formation. Additionally, the stability of these nanoparticles is improved through capping with biomolecules, which also enhances biocompatibility. This approach is not only more environmentally sustainable but also cost-effective and simpler to handle compared to traditional physical and chemical synthesis methods (Mohanta et al., 2020; Al-Dbass et al., 2022).

Plant-mediated synthesis of NPs offers several advantages, primarily due to its simplicity and eco-friendliness. The natural biomolecules in plants generally exhibit low toxicity to living cells, making them a safer and more suitable option for synthesizing AgNPs (Mittal, Chisti & Banerjee, 2013). Numerous studies have demonstrated the efficacy of plant metabolites in producing bioactive AgNPs (Soltys et al., 2021). Using plant extracts to reduce Ag^+^ ions is both faster and results in the production of more stable nanoparticles. Secondary metabolites such as flavonoids, phenolic compounds, glycosides, proteins, peptides, polysaccharides, enzymes, vitamins, and sterols serve dual roles as reducing and capping agents, enabling the eco-friendly conversion of silver ions into elemental AgNPs (Dauthal & Mukhopadhyay, 2016; Archana Sharma & Srivastava, 2020). The synergistic effects of silver metallic ions and the capping agents derived from plant extracts contribute to the remarkable biomedical effectiveness of plant-synthesized AgNPs against a variety of diseases. Recent *in vitro* studies have highlighted the intriguing biological activities of plant-derived phytochemicals and their associated nanoparticles (Gonfa et al., 2023). Green-synthesized AgNPs have proven to be highly effective antibacterial agents through various mechanisms, including the release of silver cations (Soltys et al., 2021). These cations can bind to DNA strands, cause protein damage, and disrupt bacterial cell walls, ultimately increasing reactive oxygen species (ROS) within living cells (Alduraihem et al., 2023). However, extensive research on antimicrobial resistance has revealed that antibiotic-resistant bacterial infections are primarily caused not by free-floating bacteria but by bacteria embedded within biofilms (Liu et al., 2019).

Biofilms are bacterial communities that adhere to surfaces and are encased in a self-produced extracellular polymeric substance (EPS) matrix, consisting of polysaccharides, proteins, and extracellular DNA (Altaf et al., 2021). This matrix serves as a critical survival mechanism, allowing bacteria to endure various chemical and physical stresses (Fux et al., 2005). Biofilms form a protective barrier around bacterial cells, significantly reducing antibiotic efficacy and contributing to drug resistance. Bacteria within biofilms can survive antibiotic concentrations up to 1,000 times higher than those required to affect free-floating cells (Qayyum & Khan, 2016). To combat biofilms, innovative strategies have been developed, with NPs emerging as one of the most promising solutions (Qayyum & Khan, 2016). Ag-NPs disrupt biofilm integrity by interacting with the EPS, extracellular DNA, proteins, and lipids, weakening the biofilm structure. Additionally, the generation of ROS during NP-microbe interactions damages bacterial cell envelopes, membranes, intracellular structures, and biomolecules (Qayyum & Khan, 2016). Studies have demonstrated the efficacy of AgNPs in inhibiting biofilm formation by *Escherichia coli, Enterococcus spp*., *Staphylococcus aureus*, coagulase-negative *Staphylococci spp*., *Pseudomonas aeruginosa*, and *Candida albicans* (Qayyum & Khan, 2016).

Among various types of nanoparticles, AgNPs stand out as promising anticancer agents due to their unique properties that enhance therapeutic efficacy (Jang et al., 2016). Numerous studies have demonstrated the anticancer potential of AgNPs, which penetrate cancer cells and induce cell death by activating multiple signaling pathways linked to mitochondrial dysfunction, oxidative stress, autophagy, and endoplasmic reticulum stress (Alduraihem et al., 2023).

*Punica granatum* (*P. granatum*), commonly known as pomegranate, is a deciduous small tree native to Asia and a member of the Lythraceae family. Traditionally, it has been utilized in various cultures to treat conditions such as dysentery, diarrhea, and hemorrhage. The medicinal properties of this plant are attributed to the diverse phytochemicals found in its different parts (Jang et al., 2016). Extracts from *P. granatum* have been extensively reported to exhibit therapeutic effects, including antibacterial, antiviral, antioxidant, anti-inflammatory, antihypertensive, and anticancer activities. Furthermore, the fruit is rich in antioxidants, polyphenols, and vitamins, which not only enhance its health benefits but also make it effective for reducing metal salts in nanoparticle synthesis (Kumar et al., 2018).

*Teucrium polium* (*T. polium*) and *Teucrium marum* (*T. marum*) have long been utilized in traditional herbal medicine to treat diabetes, gastric inflammation, and convulsions. These plants are native to various regions, including Southwestern Asia, North Africa, Eastern Europe, and the western Mediterranean islands (Firoznezhad et al., 2022). Extracts from *T. polium* and *T. marum* are well-known for their diverse therapeutic effects, which include anti-ulcer, anti-inflammatory, antibacterial, antihypertensive, antinociceptive, and anticancer activities (Hashemi, Tasharrofi & Saber, 2020). The primary phytochemical constituents of *Teucrium* species-such as diterpenoids, flavonoids, iridoids, sterols, and terpenoids-are believed to play a significant role in their cytotoxic and antitumor properties, making these plants a valuable resource for biomedical research and applications (Hashemi, Tasharrofi & Saber, 2020).

Although the individual therapeutic properties and phytochemical profiles of *P. granatum* and *Teucrium* species have been well-documented, their application in the biosynthesis of AgNPs remains underexplored. This study aimed to investigate the potential of extracts from *P. granatum*, *T. polium* and *T. marum* (collected from southern Tunisia) for synthesizing silver nanoparticles AgNPs. Due to their rich phytochemical composition, particularly polyphenols, flavonoids, and terpenoids with a strong antimicrobial, antioxidant, and anti-inflammatory properties, which serve as natural reducing and stabilizing agents in nanoparticle formation, the rich phytochemical antioxidants, present a promising pathway for sustainable nanoparticle synthesis. The resulting AgNPs were characterized using various analytical techniques, such as X-ray diffraction (XRD) and dynamic light scattering (DLS). The inherent biological properties of these plants, particularly their antibacterial and anticancer activities, further enhance their potential for biomedical applications. By leveraging these plant extracts for nanoparticle synthesis, this study aimed to develop eco-friendly, biologically active silver oxide nanoparticles with promising therapeutic applications, particularly in drug delivery and cancer therapy.

## Materials & Methods

### Preparation of the plant extracts

Fresh leaves of *T. polium* and *T. marum*, collected in March 2023 from the Kasserine region of Tunisia, along with *P. granatum* bark from the Gabes region of Tunisia, were utilized in this study. The plant tissues were first dried and ground into a fine powder. Ethanolic extracts were then prepared by mixing equal volumes of ethanol and water (50 mL) with the powdered plant material (15 mg). The mixture was heated at 100 °C for 1 h, and the homogenate was filtered to obtain the final extract.

### Green synthesis and characterization of AgNPs

The peel *P. granatum* and the leaves of *T. polium* and *T. marum* were carefully dried and subjected to boiling in ethanol to facilitate the extraction of phytochemicals. The resulting mixtures were then filtered to obtain the purified extract. Then, they were individually mixed with a 6 mM silver nitrate solution (AgNO_3_) and incubated at 60 °C with shaking in an orbital shaker according to the protocol of Qamar et al. (2020) and Qamar et al. (2020). The homogenized solution was centrifuged at 7,000 rpm for 10 min, and the pellet was washed with ethanol and dried at 600 °C for 2 h using high-temperature drying oven, resulting in a change in color from yellow to dark brown (Vorobyova et al., 2022). The color change from yellow to dark brown in the reaction mixture serves as a visual indication of nanoparticle formation, attributed to the surface plasmon resonance of the synthesized AgNPs. Surface plasmon resonance refers to the collective oscillation of conduction band electrons at the surface of the synthesized silver nanoparticles.

### Characterization of AgNPs

To thoroughly assess the structural, optical, and functional properties of the synthesized AgNPs—key factors in determining their potential applications—a detailed and comprehensive characterization was performed.

#### Structural analysis

In this study, X-ray diffraction (XRD) was employed to assess the structural properties of the synthesized AgNPs. As a non-destructive technique, XRD provides critical insights into both the crystallite size and the overall nanoparticle structure. X-ray diffraction analysis was performed using a D8 Advance X-ray diffractometer, equipped with a graphite monochromator and a CuK*α* radiation source (*λ* = 1.5418 Å), operating at 45 kV and 40 mA. Crystallite size was determined using the Debye-Scherrer equation D = k*λ*/ *β*cos *θ*, ensuring precise evaluation of nanoparticle dimensions. In this equation: D represents the crystallite size; *λ* is the wavelength of the X-ray source; *β* is the full width at half maximum (FWHM) of the diffraction peak (in radians); k the Scherrer constant, typically ranges from 0.9 to 1; and *θ* is the Bragg angle (in radians) (Singh et al., 2018).

#### Optical properties

The optical properties of the synthesized AgNPs were assessed using UV-Vis spectroscopy (Shimadzu-1800, Kyoto, Japan). The absorption of radiation occurs in both the visible range (400–800 nm) and the ultraviolet range (200–400 nm). It is widely recognized that silver nanoparticles exhibit a yellowish-brown color. Further confirmation of this observation was obtained using a UV-Vis spectrophotometer. The UV spectra show absorption band which correspond to the surface plasmon absorption of the AgNPs. This absorption band is also a result of the oscillation of free conduction electrons within the AgNPs, triggered by the absorption of electromagnetic radiation. Furthermore, this technique was employed to determine the optical band gap energy (Eg) of the biosynthesized silver nanoparticles, providing valuable insights into their electronic structure and potential applications.

#### Infrared spectroscopy

Fourier Transform Infrared Spectroscopy (FTIR) is a powerful analytical technique that identifies functional groups in a sample by analyzing its interaction with infrared radiation. In the synthesis of AgNPs, FTIR offers several benefits such the identification of the molecules that contribute to the reduction and stabilization of the nanoparticles, the determination of the functional groups that facilitate the binding of biomolecules to the nanoparticles, and the assessment of the chemical stability of the nanoparticles over time or under varying environmental conditions. When analyzing FTIR spectra to identify functional groups, it is crucial to consider several key factors. Examining spectral regions can aid in identifying the functional groups within a molecule. Additionally, the intensity of the absorption peaks may differ based on the concentration of the functional group and the strength of the interactions between molecules. Moreover, the shifting of absorption peaks can occur due to solvation effects, molecular interactions, or alterations in molecular structure.

In this study, FTIR spectroscopy (Shimadzu IR, Prestige 21, Nakagyo-Ku, Japan) was performed to characterize the functional groups of synthesized AgNPs (Almayouf et al., 2024). Additionally, FTIR was used to monitor conformational changes and chemical group interactions during vancomycin loading onto the surface of AgNPs, within the wavelength range of 4,000–400 cm^−^^1^ (Jadoun et al., 2021).

#### Particle size analysis

DLS is a commonly employed technique for analyzing the size distribution, polydispersity index (PDI), and zeta potential of nanoparticles in suspension. It assesses the Brownian motion of particles within a liquid medium and correlates it with their hydrodynamic diameter using the Stokes-Einstein equation. The PDI serves as a measure of the uniformity of particle size distribution. In this study, the DLS analysis was conducted using a Zetasizer (HT Laser, ZEN3600, Malvern Nano series) from Malvern Instruments (Worcestershire, UK), to determine the hydrodynamic sizes (Almayouf et al., 2024) and correlation coefficients of the synthesized AgNPs. The measurements were performed at 25 °C using water as the dispersion medium. The analysis was carried out under the following conditions: refractive index (RI) of 1.33, dispersion viscosity of 0.887 cP, and dielectric constant of 78.5. These parameters ensured accurate characterization of nanoparticle size distribution and stability in the aqueous medium.

### Antibacterial activity

Two Gram-positive strain *Staphylococcus epidermidis* (*S. epidermidis*) and *Staphylococcus aureus* (*S. aureus*) and three Gram-negative strains *Escherichia coli* (*E. coli*), *Pseudomonas aeruginosa* (*P. aeruginosa*), and *Serratia marcescens (S. marcescens*) were used. The antimicrobial activity of AgNPs (Tp-AgNPs, Tm-AgNPs, and Pg-AgNPs) was evaluated using the well diffusion method on Muller Hinton, as described by Adnan et al. (2022) and Adnan et al. (2022). Petri dishes containing Mueller-Hinton Agar (MHA) agar were uniformly inoculated with various bacterial cultures, and 100 µL of AgNPs was added to each well. The plates were incubated at 37 °C for 24 h. The inhibition zones were measured and compared against a control and the standard antibiotic chloramphenicol (2.5 mg/mL) to assess the inhibitory effect of AgNPs on bacterial growth. The antimicrobial assay was performed in triplicate, and the inhibition zones were measured for each replicate.

In the second phase, the antimicrobial activity of AgNPs was further analyzed by determining the minimum inhibitory concentration (MIC) and minimum bactericidal concentration (MBC) for bacteria treated with varying concentrations of AgNPs. Bacterial strains were cultured in Mueller Hinton Broth (MHB) for 24 h at either 37 °C or 30 °C, depending on the strain’s requirements. AgNPs were dissolved in Mueller-Hinton Broth containing 10% DMSO and added at a volume of 100 µL per well. A serial 1:2 dilution was performed by transferring 100 µL from one well to the next. Each well was then supplemented with 80 µL of MHB and 20 µL of bacterial inoculum. The first two wells served as controls: the negative control contained 180 µL of MHB without AgNPs and the positive control contained 100 µL of Chloramphenicol (2.5 mg/mL) mixed with 80 µL of MHB. After 24 h of incubation, 20 µL of MTT solution (one mg/mL) was added to each well to evaluate bacterial viability and proliferation, following the method described by Alfehaid et al. (2024) and Alfehaid et al. (2024). The MIC, expressed in mg/mL, was determined as the lowest concentration of AgNPs that prevented bacterial growth after 24 h. To determine the MBC, 10 µL aliquots from wells showing no visible growth were plated on MHA agar. The MBC was identified as the lowest concentration of AgNPs that caused at least 99.9% bacterial lysis after 24 h of incubation.

### Anti-biofilm activity

The anti-biofilm activity of Tp-AgNPs, Tm-AgNPs, and Pg-AgNPs was assessed using a 96-well flat-bottom polystyrene microtiter plate, following the method described by Abdelli et al. (2019) and Abdelli et al. (2019). The anti-adhesive activity was tested against two strong biofilm-forming model strains, *P. aeruginosa* and *S. aureus*.

Each well of the plate was filled with 100 µL of Tryptic Soy Broth (TSB), adjusted to achieve a final optical density (OD) of 0.1 at 600 nm. Subsequently, 100 µL of AgNPs dissolved in TSB containing 10% DMSO was added to achieve final concentrations of 0.039, 0.078, 0.156, 0.312, 0.625, 1.25, 2.5, and five mg/mL. Control wells contained only TSB supplemented with 2.25% glucose and 20% (v/v) DMSO, along with the bacterial suspension. After incubating the plates at 30 °C for *S. aureus* and 37 °C for *P. aeruginosa* for 24 h, the well contents were discarded by inverting the plates. Each well was then gently rinsed twice with 200–250 µL of sterile phosphate-buffered saline (PBS; 137 mM NaCl, 2.7 mM KCl, 10 mM Na_2_HPO_4_, 1.76 mM KH_2_PO_4_; pH 7.2) to remove planktonic cells (Abdelli et al., 2019). Following the washing steps, the plates were dried at 60 °C for 1 h. Each well was then treated with 150 µL of a 0.2% (w/v) crystal violet solution prepared in 20% ethanol and incubated at room temperature for 15 min, following the method described by Vasudevan et al. (2003) and Vasudevan et al. (2003). After staining, the crystal violet solution was discarded, and unfixed dye was removed by washing the wells three times with sterile water. To solubilize the bound dye, 200 µL of 33% glacial acetic acid was added to each well, and the plates were incubated at room temperature for 1 h. The OD was measured at 570 nm using a Varioskan microplate reader (Thermo Fisher Scientific, Waltham, MA, USA). The percentage of adhesion inhibition was calculated using the formula (1): [(*OD*(*control*) − *OD*(*treated* *strain*))÷*OD*(*control*)] × 100, where control was the wells containing bacterial strains without AgNPs.

### Biofilm eradication

The effect of nanoparticles (Tp-AgNPs and Tm-AgNP) on the biofilm formed by *S. aureus* was investigated using the method described by Tsukatani, Sakata & Kuroda (2020) and Tsukatani, Sakata & Kuroda (2020), with minor modifications. Biofilm formation was carried out over 48 h in 96-well plates. Following incubation and biofilm formation, the medium and non-adherent bacteria were removed by aspiration, followed by two washes with PBS. A volume of 200 µL containing AgNPs at concentrations 0.039, 0.078, 0.156, 0.312, 0.625, 1.25, 2.5 and 5 mg/mL was added to the wells. The plates were then re-incubated at 30 °C for 24 h. The biofilms were then stained with crystal violet, as described previously. A control without the addition of the anti-biofilm agent was included. The percentage of biofilm eradication was calculated by comparing the OD values (570 nm) of the control and the treated samples using the following equation (1): [(*OD*(*control*) − *OD*(*treated* *strain*))÷*OD*(*control*)] × 100 where the control was the untreated strain with AgNPs.

### Cytotoxicity assays

The MCF-7 breast cancer cell line, obtained from the American Type Culture Collection (ATCC), was cultured in Dulbecco’s Modified Eagle’s Medium (DMEM) supplemented with 10% fetal bovine serum (FBS), 50 IU/mL penicillin, and 50 µg/mL streptomycin. The cells were seeded into 96-well plates and grown to approximately 40% confluence. Tested NPs (Tp-AgNPs, Tm-AgNPs, and Pg-AgNPs) were added at concentrations ranging from 0.039 to five mg/mL and incubated for 48 h at 37 °C in a humidified atmosphere containing 5% CO_2_. Cell viability was evaluated using the MTT assay as described by Abdelli et al. (2019) and Abdelli et al. (2019). After AgNPs treatment, the medium was replaced with fresh medium, and 10 µL of MTT solution (five mg/mL in PBS) was added to each well. Following a 4-h incubation period, 100 µL of 10% sodium dodecyl sulfate (SDS) solution was added to each well to dissolve the formazan crystals. OD was measured at 570 nm using a Varioskan microplate reader (Thermo Fisher Scientific, Waltham, MA, USA). Cell growth inhibition was calculated using the formula (2): (%) *cell* *survival* = (*AT*÷*AO*) × 100, where A0: control absorbance and AT: treated cells absorbance.

## Results and discussion

### Structural parameters of AgNPs

#### Analysis of XRD

**Figure 1:**
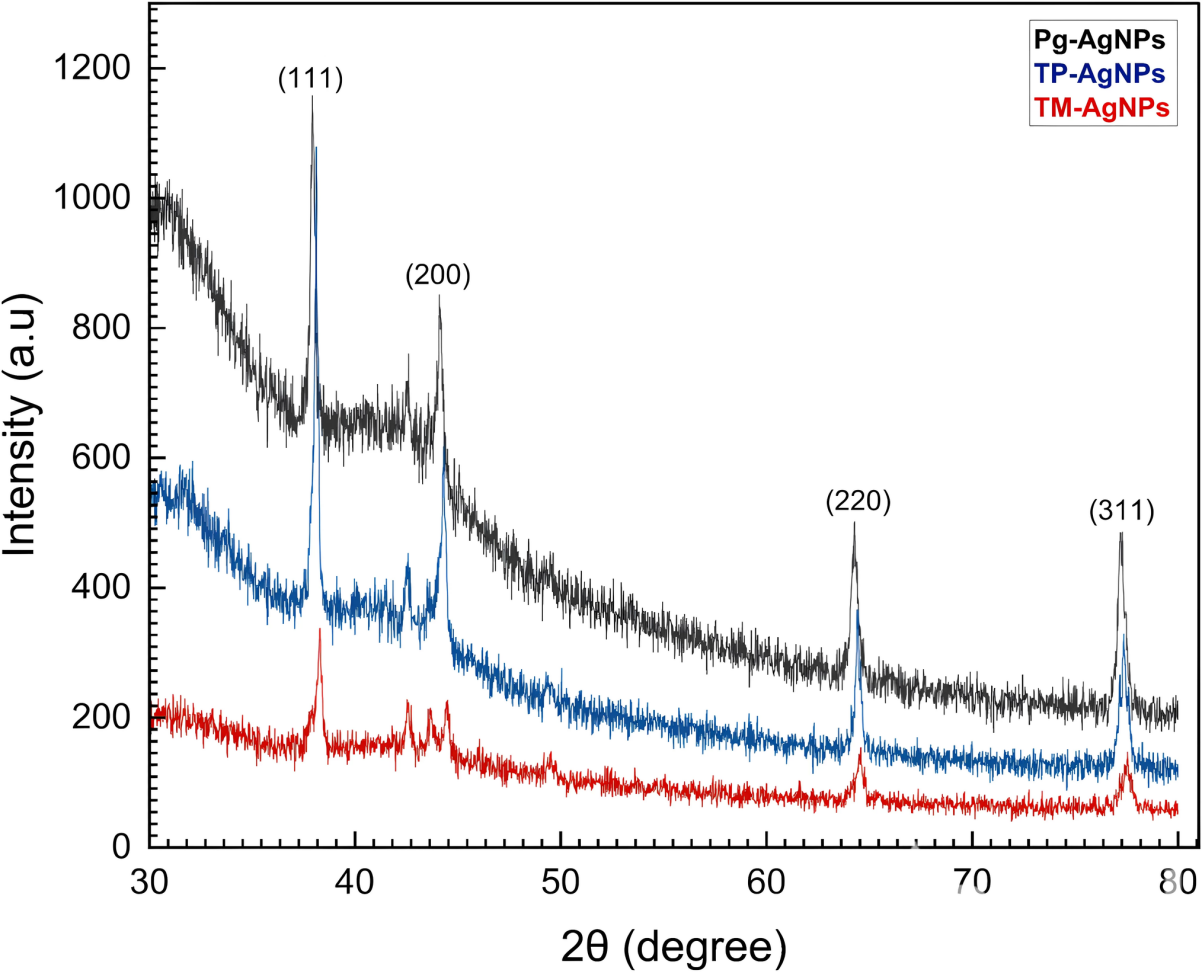
XRD patterns of Tp-AgNPs, Tm-AgNPs, and Pg-AgNPs.

The XRD analysis of the biosynthesized AgNPs revealed four prominent peaks, corresponding to the (111), (200), (220), and (311) crystal planes (Fig. 1). Utilizing the PANalytical X’Pert High Score Plus Software, these peaks verified the face-centered cubic (fcc) crystal structure of the AgNPs, as indicated by JCPDS file No. 03-065-2871 for Tp-AgNPs and Pg-AgNPs, and JCPDS file No. 01-087-0718 for Tm-AgNPs. The experimental diffraction angles closely aligned with the standard values, further validating the crystal structure. The stability of the AgNPs could be attributed to the presence of multiple reducing agents in the solution, which also influenced their crystalline characteristics-a phenomenon widely reported in biosynthesized NPs (Khane et al., 2022). Additionally, unidentified peaks in the diffraction pattern may indicate the presence of bioorganic compounds on the AgNPs surfaces. The size of the AgNPs plays a significant role in shaping the XRD peaks. The broader diffraction profiles, compared to bulk silver, suggested the formation of larger AgNPs. These observations highlighted the impact of biosynthesis conditions on the structural properties of AgNPs.

The interplanar d-Spacing and the lattice parameters for Tp-AgNPs, Tm-AgNPs, and Pg-AgNPs, which are tabulated in Table 1, were derived based on observed diffraction patterns. The estimated values were found to be in close agreement with the standard values of *d* = 2.35911 Å and *a* = 4.0861 Å for the crystal plane (111), which have been commonly reported in previous studies, specifically JCPDS file No. 03-065-2871 for Tp-AgNPs and Pg-AgNPs, as well as JCPDS file No. 01-087-0718 for Tm-AgNPs (with *d* = 2.35403 Å and *a* = 4.0773 Å). The size of the AgNPs’ crystallites was also calculated using Scherer’s equation D = K *λ*/*β* cos*θ* 18, where D represent the crystallites size (nm), *K* = 0.9 the Scherrer constant, *λ* = 0.15406 nm was the wavelength of the X-ray sources, *β* represents the full width at half maximum (FWHM in radians) of the main peak, and *θ* was the peak position in radians (Table 1).

#### DLS analysis

**Figure 2:**
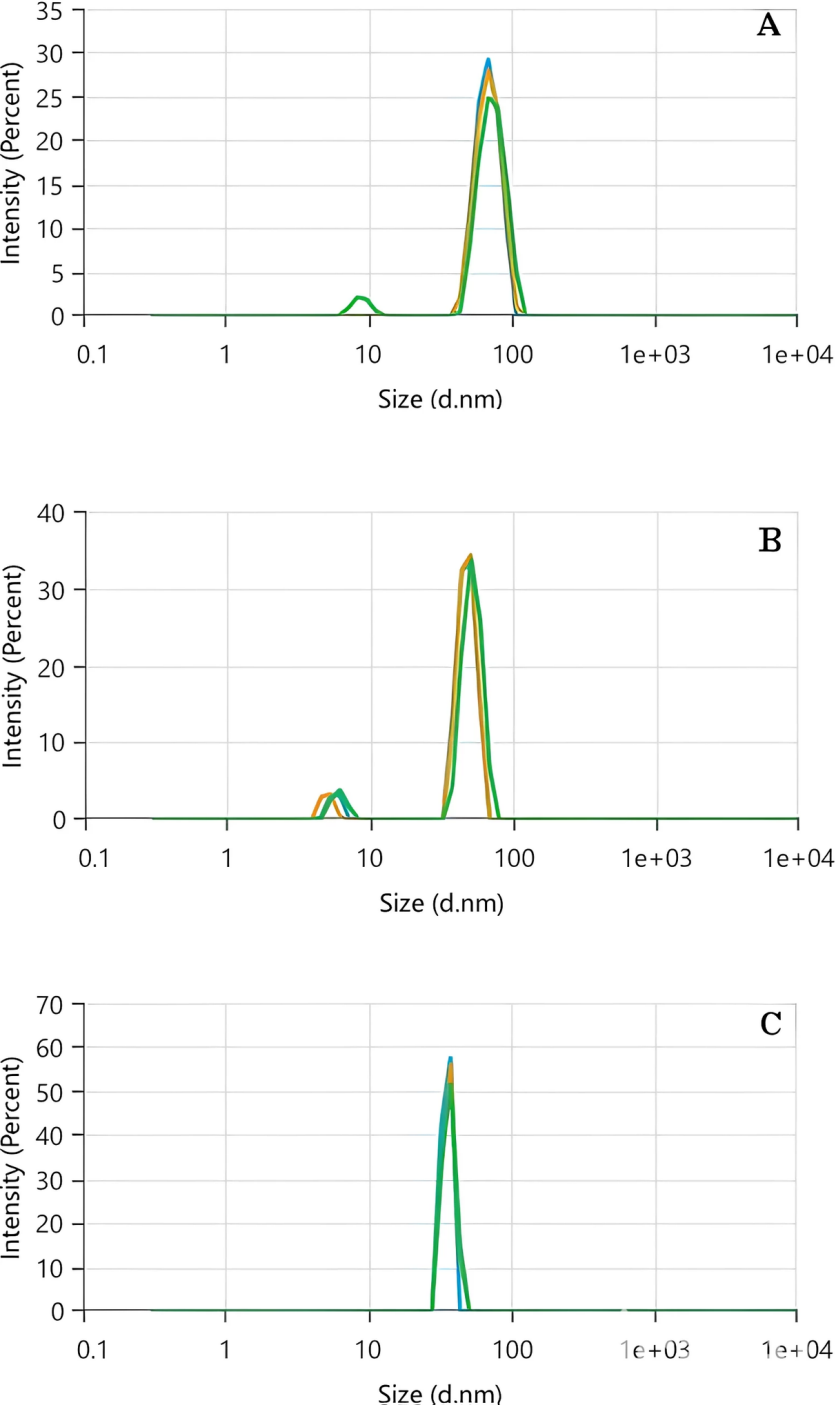
DLS analysis of Tp-AgNPs (A), Tm-AgNPs (B), and Pg-AgNPs (C) compounds.

DLS is a highly versatile technique for measuring and analyzing NP size. The method is based on detecting fluctuations in light scattered by dispersed NPs, which result from their random motion, known as Brownian motion. These fluctuations are directly influenced by the hydrodynamic diameters of the NPs, providing insights into their size distribution. A critical parameter in DLS analysis is the polydispersity index (PDI), a dimensionless value that evaluates the uniformity of particle size distribution and the extent of NP aggregation. The PDI ranges from 0 to 1, with values offering important clues about the sample; a PDI ≤ 0.5 indicates a primarily monodisperse and homogeneous particle distribution while a PDI > 0.7 suggests a highly polydisperse system, characterized by a wide range of particle sizes. This distinction makes PDI a key factor in assessing the quality and stability of NPs in a given dispersion (Danaei et al., 2018). The Zeta size as a function of the intensity (%) for Tp-AgNPs, Tm-AgNPs, and Pg-AgNPs were shown in Figs. 2A, 2B and 2C, respectively. The particle size, the PDI, and the intercept value were determined for all samples (Table 2). The results indicated that both Tp-AgNPs, Tm-AgNPs exhibited monodisperse distributions and demonstrated long-term stability. In contrast, the Pg-AgNPs showed a wide range of particle sizes, suggesting that these NPs were semi-uniform in nature. This variation in size distribution highlighted the differences in the synthesis processes and the resulting stability of the NPs.

**Table 1:** X-ray structural parameters of Tm-AgNPs, Tp-AgNPs, and Pg-AgNPs extracts.

Silver nanoparticles	2θ(°)	Interplanar spacing d (Å)	Lattice parameters (Å)	FWHM (°)	Crystallite size (nm)
Tm-AgNPs	38.206	2.354	4.077	0.580	14.492
Tp-AgNPs	38.103	2.359	4.086	0.299	28.125
Pg-AgNPs	37.936	2.369	4.099	0.272	30.844

**Table 2:** Particle size, PDI, and the intercept parameters of Tp-AgNPs, Tm-AgNPs, and Pg-AgNPs.

Silver nanoparticles	Z-average (nm)	Polydispersity index	Intercept
Tp-AgNPs	198	0.222	0.850
Tm-AgNPs	343	0.392	0.904
Pg-AgNPs	734	0.703	1.024

The XRD patterns indicated that the size of all three AgNPs was smaller than their hydrodynamic diameters. This discrepancy could be attributed to the fact that XRD analysis measures the crystallite size, which reflects the dimensions of the crystalline regions within the NPs. In contrast, the hydrodynamic diameter, as determined by techniques like DLS, accounted for the overall size of the particle, including both the nanoparticle core and anysurrounding solvation shell or macromolecular components. Therefore, the hydrodynamic diameter typically appeared larger than the crystallite size observed in XRD analysis (Stetefeld, McKenna & Patel, 2016).

As previously stated, DLS analysis evaluates the strength of scattered light by particles present in a liquid or suspension to establish their size distribution. The correlation coefficient, also known as the autocorrelation function, plays a crucial role in DLS analysis. It offers insights into the movement of particles over a period, enabling the estimation of their size. The behavior of the autocorrelation function reflects the variations in scattered light intensity over time. In the case of monodisperse systems, where all particles are of the same size, the autocorrelation function typically demonstrates a gradual decrease following an exponential pattern. However, for polydisperse systems with particles of varying sizes, the decay of the autocorrelation function becomes more intricate and can be represented by a combination of exponential terms. The rate of decay in the autocorrelation function is directly linked to the diffusion coefficient of the particles. Smaller particles exhibit faster diffusion, resulting in a more rapid decline in correlation. Conversely, larger particles diffuse at a slower pace, resulting in a more gradual decay of the correlation.

**Figure 3:**
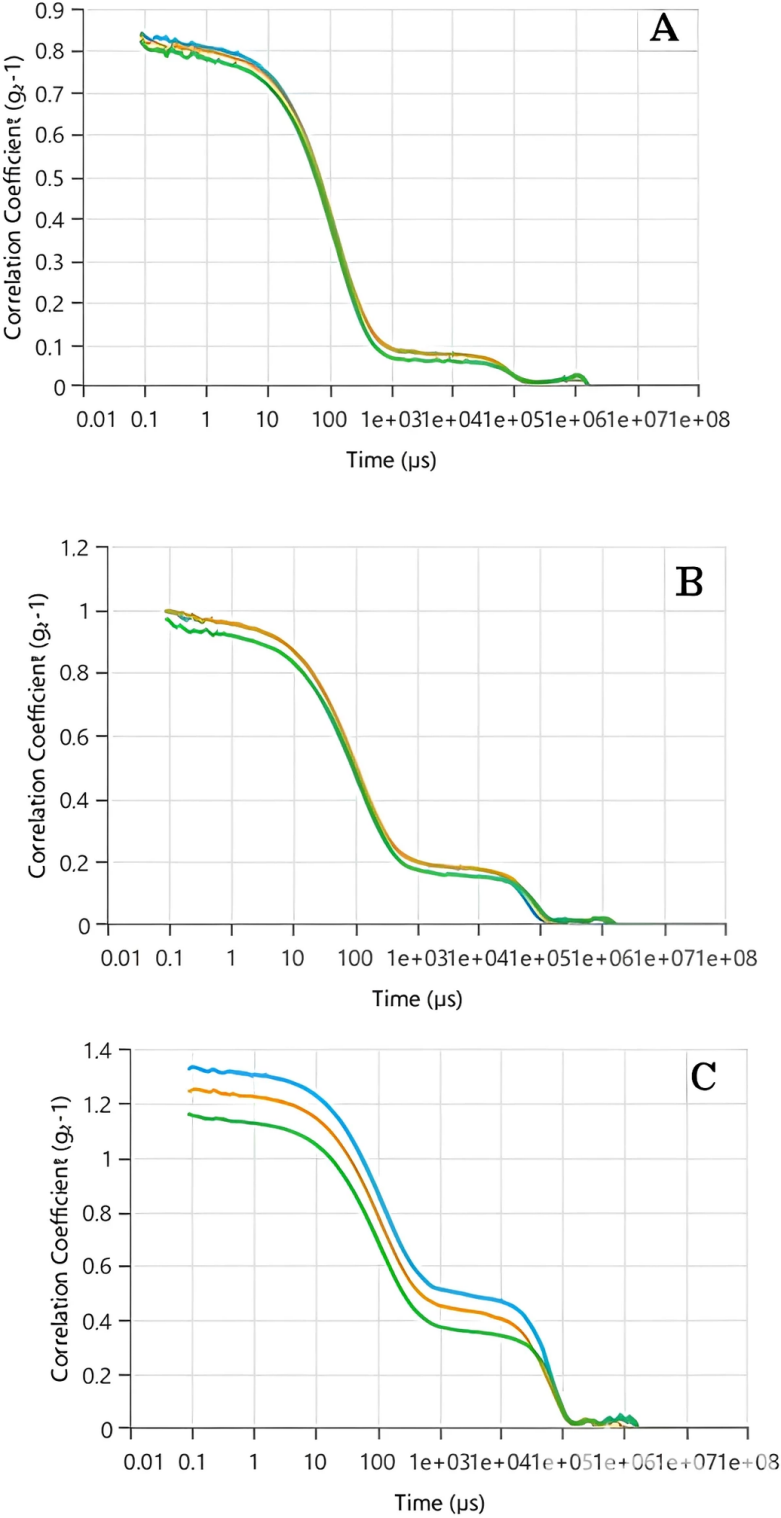
The autocorrelation functions of Tp-AgNPs of Tp (A), Tm-AgNPs (B), and Pg-AgNPs (C) compounds.

According to the data presented in Figs. 3A–3C, the correlation coefficient peaks at *t* = 0, indicating a strong initial correlation between the scattered light intensity and its subsequent values. As time progressed, the correlation coefficient decreased, with the rate of decay influenced by the particle size. For Tp-AgNPs and Tm-AgNPs (Figs. 3A and 3B, respectively), the autocorrelation function showed a rapid decay, suggesting the presence of smaller particles with higher diffusion coefficients. In contrast, the slower decay observed in the autocorrelation function of Pg-AgNPs (Fig. 3C) pointed to larger particles with lower diffusion coefficients. These observations were consistent with the earlier results from the Zeta size measurements, which also indicated intensity variations corresponding to particle size.

#### Analysis of UV-vis spectra

**Figure 4:**
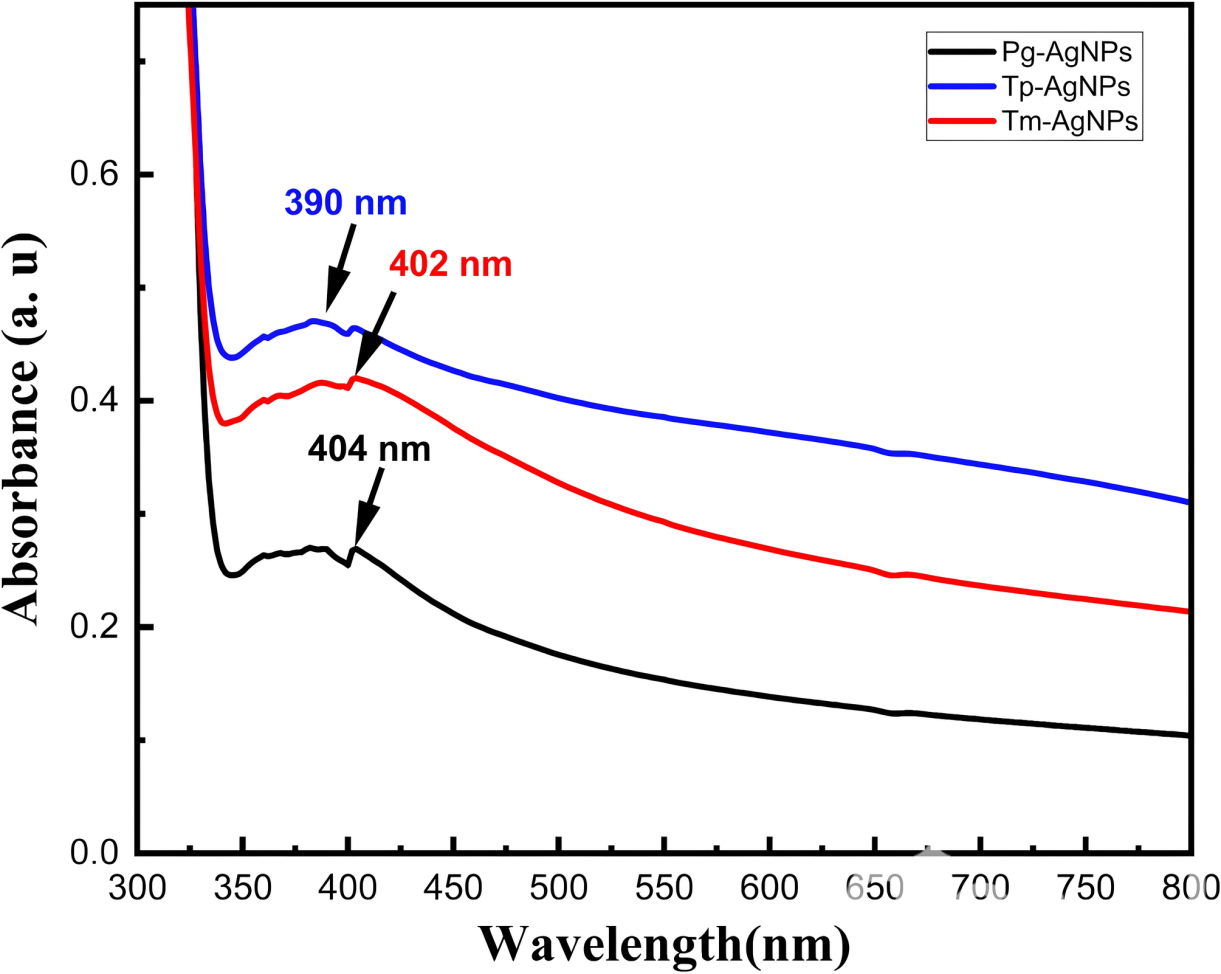
UV spectra of Tp-AgNPs, Tm-AgNPs, and Pg-AgNPs extracts.

Figure 4 illustrated the UV–vis spectra for the extracts of Tm-AgNPs, Tp-AgNPs, and Pg-AgNPs. The UV spectra showed absorption bands at 390 nm, 402 nm, and 404 nm for Tp-AgNPs, Tm-AgNPs, and Pg-AgNPs, respectiveley, which were indicative of the surface plasmon resonance of the AgNPs. These absorption bands arised from the collective oscillations of the free electrons at the surface of the NPs. The biosynthesis process was monitored visually by noting the color change, which signified the reduction of AgNO_3_ and the subsequent formation of AgNPs. The results demonstrated that the synthesized samples produced AgNPs with unique physiochemical properties, as reflected in their distinct absorption spectra. Furthermore, the absorption peaks of Tp-AgNPs and Tm-AgNPs exhibited significantly higher intensities compared to Pg-AgNPs, suggesting a tendency for monodispersity. This observation was in excellent agreement with the DLS analysis, which further corroborated the uniform size distribution of these NPs. The optical band gaps (Eg) of Tp-AgNPs, Tm-AgNPs, and Pg-AgNPs were calculated using the equation Eg = 1,240/*λ*_max_ (Awad et al., 2021), yielding values of 3.18 eV, 3.08 eV, and 3.07 eV, respectively. Notably, these Eg values were within a suitable range for optical applications, suggesting that these biosynthesized AgNPs had potential for use in optoelectronic devices.

#### Analysis of FTIR spectra

By employing FTIR spectroscopy to analyze the AgNPs, we confirmed the presence of specific functional groups and substantiated the plant extract’s role as both a reducing and capping agent. Variations in peak intensity or shifts in wavenumber suggested the involvement of these functional groups in the binding processes.

**Figure 5:**
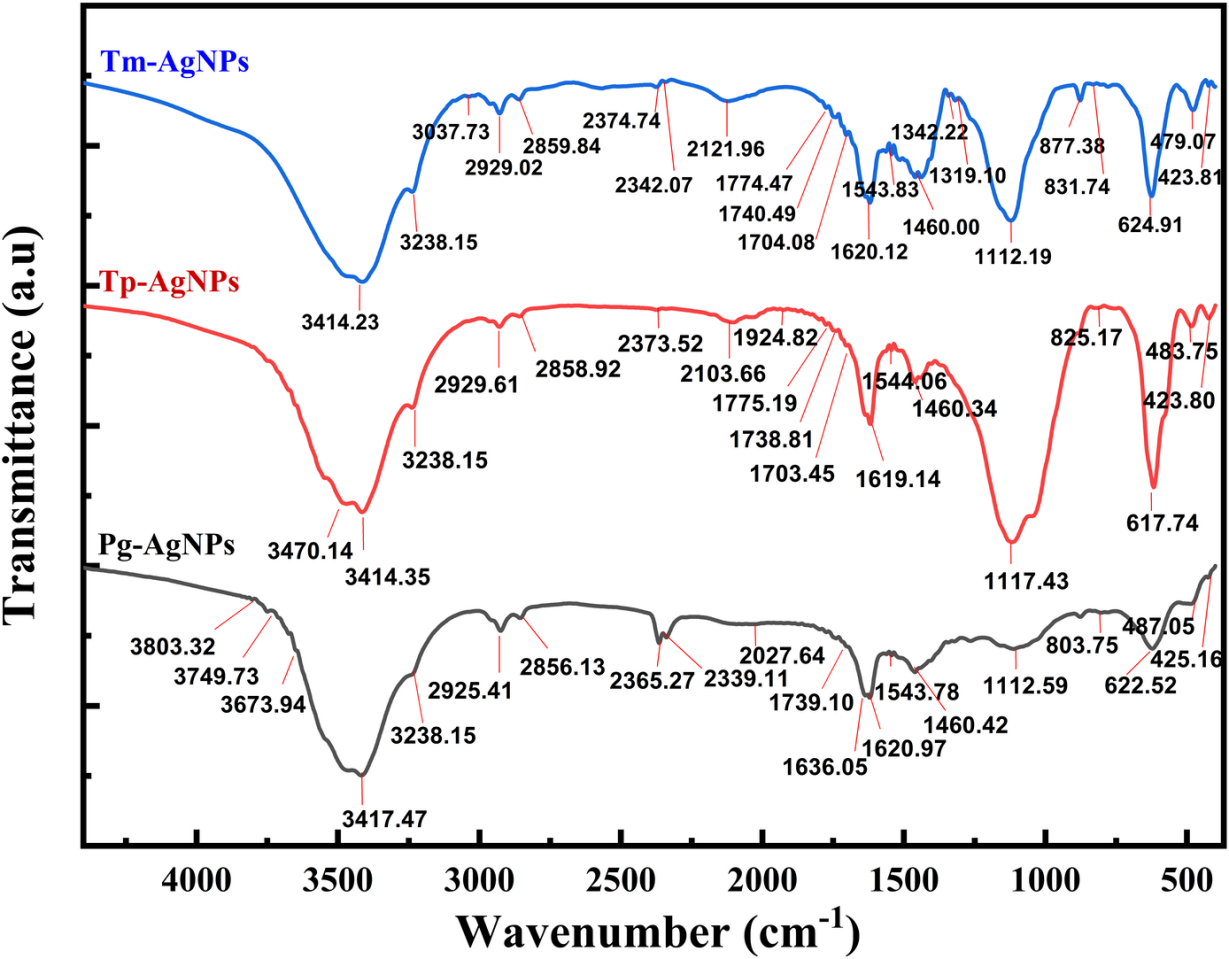
FTIR spectra of Tp-AgNPs, Tm-AgNPs, and Pg-AgNPs extracts.

The FTIR spectrum of Tm-AgNPs (Fig. 5) displayed absorption bands at the following wavelengths: 3,414.23, 3,238.15, 3,037.73, 2,929.02, 2,859.84, 2,374.74, 2,342.07, 2,121.96, 1,774.47, 1,740.49, 1,704.08, 1,620.12, 1,543.83, 1,460.00, 1,342.22, 1,319.10, 1,112.19, 877.38, 831.74, 624.91, 479.07, and 423.81 cm^−^^1^, while the FTIR spectrum of Tm-AgNPs showed absorption bands at 3,470.14, 3,414.35, 3,238.15, 2,929.61, 2,858.92, 2,373.52, 2,103.66, 1,775.19, 1,738.81, 1,703.45, 1,619.14, 1,544.06, 1,460.34, 1,117.43, 825.17, 617.74, 483.75, and 423.80 cm^−^^1^. Figure 5 also depicted the FTIR spectrum of Pg-AgNPs which revealed absorption bands at 3,803.32, 3,749.73, 3,673.94, 3,417.47, 3,238.15, 2,929.41, 2,856.13, 2,365.27, 2,339.11, 2,027.64, 1,739.10, 1,636.05, 1,620.97, 1,543.78, 1,460.42, 1,112.59, 803.75, 622.52, 487.05, and 425.16 cm^−^^1^. There was a slight difference in the binding sites of the synthesized AgNPs, as indicated by their IR spectra. Shifts in absorption peaks within an IR spectrum could result from various factors, including solvation effects, which suggest that interactions between the molecule and solvent may lead to these shifts. This can occur due to polarization effects or hydrogen bonding. Additionally, molecular interactions—such as hydrogen bonds, van der Waals forces, or π − π interactions—can also contribute to shifts in absorption peaks (Almayouf et al., 2024).

The bands detected in the IR spectra at approximately 3,238, 3,400, 2,929, 2,860, 1,620, 1,460, and 1,112 cm^−^^1^ suggested the presence of various functional groups. The peaks at around 3,238 and 3,414 cm^−^^1^ were likely associated with the stretching vibrations of the hydroxyl group (-O–H), a hallmark of alcohols. The bands at 2,929 and 2,860 cm^−^^1^ might correspond to the stretching of the N–H bond typical of amines, as well as indicating the -CH stretching associated with alkyl groups. Furthermore, the band near 1,620 cm^−^^1^ could represent the C=O bond found in carboxylic acids or esters, while the peak at 1,460 cm^−^^1^ was likely indicative of the amide I bond (N–C=O) present in proteins, esters, or ethers. Lastly, the absorption band at 1,112 cm^−^^1^ might highlight the C–N bond found in aliphatic amines or in alcohols and phenols. Additionally, the emergence of new peaks at around 877, 830, and 803 cm^−^^1^ suggested the potential attachment of CH groups to AgNPs. These interpretations aligned with findings from previous research (Manikandan et al., 2017).

### Biological studies of silver nanoparticles

#### Antibacterial effect

##### Agar well diffusion assay.

**Figure 6:**
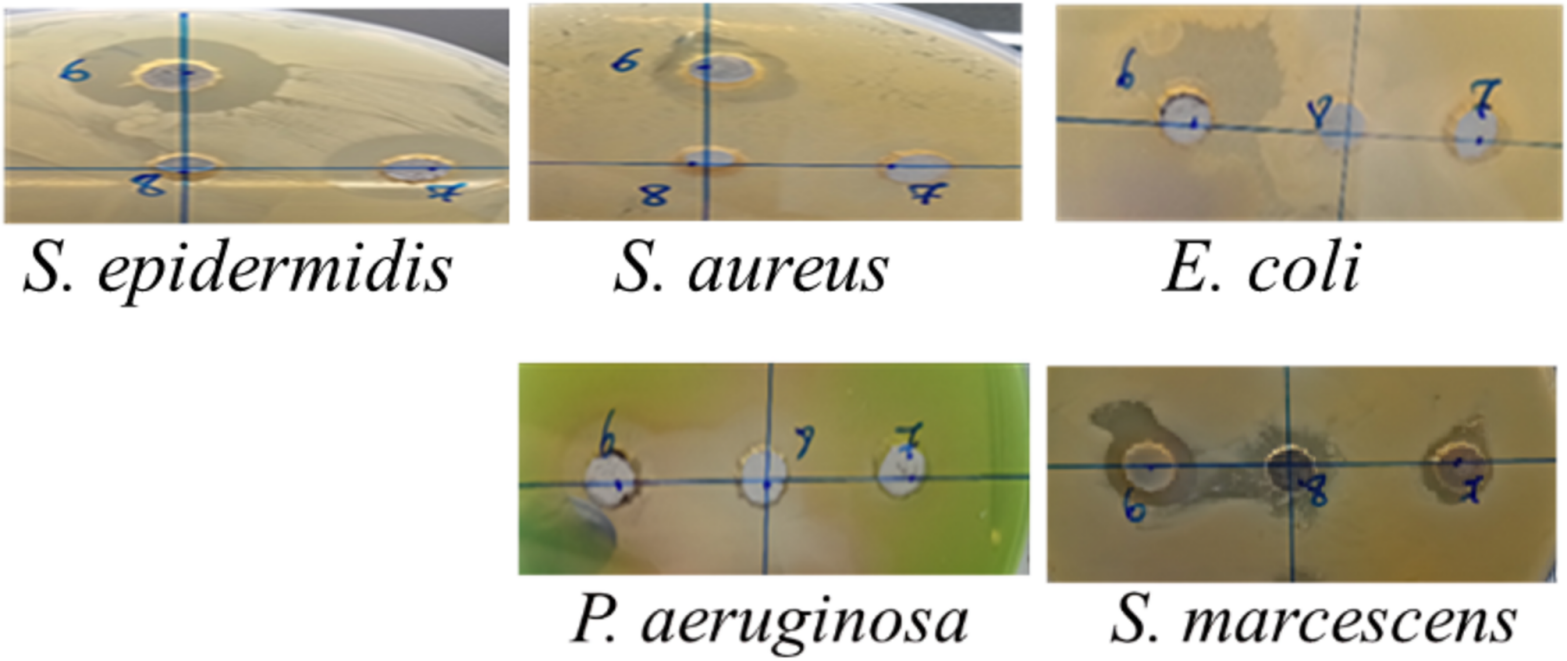
The inhibition zone diameters of Tp-AgNPs of Tp (6), Tm-AgNPs (7), and Pg-AgNPs (8) against gram positive and negative bacteria.

The inhibition zone diameters demonstrated varying antibacterial effects of the different AgNPs (Tp-AgNPs, Tm-AgNPs, and Pg-AgNPs) against both Gram-positive and Gram-negative bacteria (Table 3). These effects were compared to chloramphenicol, which produced larger inhibition zones ranging from 30 to 35 mm across the bacterial strains. For the Gram-positive strain *S. epidermidis*, Tp-AgNPs and Tm-AgNPs demonstrated significant antibacterial activity, producing inhibition zones of 20 mm and 19.5 mm, respectively, though these were smaller than those observed for chloramphenicol (Fig. 6). In contrast, Pg-AgNPs exhibited no visible antibacterial effect when evaluated using the agar well diffusion method (Table 3). *S. aureus* exhibited moderate susceptibility, with inhibition zones of 11.5 mm and 12.5 mm for Tp-AgNPs and Tm-AgNPs, respectively, while Pg-AgNPs showed no effect. Among Gram-negative strains, *E. coli* and *S. marcescens* displayed mild inhibition zones of 14 mm and 10 mm with Tp-AgNPs and Tm-AgNPs, respectively, and a 10 mm inhibition zone for Pg-AgNPs. For *P. aeruginosa*, Tp-AgNPs produced a notable inhibition zone of 19 mm, which was significantly reduced for Tm-AgNPs (10 mm) and Pg-AgNPs (15 mm) (Fig. 6). In contrast, Tm-AgNPs showed only a 10 mm zone, and Pg-AgNPs exhibited no inhibition (Table 3).

**Table 3:** Inhibition zone diameters, minimum inhibitory concentration MIC and minimum bactericidal concentration MCB values of AgNPs (Tp-AgNPs, Tm-AgNPs, and Pg-AgNPs) against Gram-positive and Gram-negative bacteria.

**Strains**	**Gram**	**Tp-AgNPs**	**Tm-AgNPs**	**Pg-AgNPs**	**Chloramphenicol**
**Inhibition zone** ** diameter** ** (mm)**					
*Staphylococcus epidermidis*	+	20 ± 0	19.5 ± 0.7	0	30 ± 0
*Staphylococcus aureus*	+	11.5 ± 0.7	12.5 ± 0.7	0	32 ± 0
*Escherichia coli *	–	14 ± 0	10 ± 0	10	35 ± 0
*Pseudomonas aeruginosa*	–	19 ± 0	10 ± 0	15	30 ± 0
*Serratia marcescens*	–	14.5 ± 0.7	10 ± 0	0	35 ± 0
**MIC (mg/mL)**					
**Strains**	**Gram**	**Tp-AgNPs**	**Tm-AgNPs**	**Pg-AgNPs**
*Staphylococcus epidermidis*	+	0.15	5	5
*Staphylococcus aureus*	+	0.15	5	5
*Escherichia coli*	–	0.31	5	1.25
*Pseudomonas aeruginosa*	–	0.039	1.25	2.5
*Serratia marcescens*	–	0.31	5	0
**MBC (mg/mL)**					
*Staphylococcus epidermidis*	+	0.31	5	5
*Staphylococcus aureus*	+	2.5	5	5
*Escherichia coli*	–	0.62	5	1.25
*Pseudomonas aeruginosa*	–	0.62	1.25	2.5
*Serratia marcescens*	–	0.62	5	5

These findings indicated that Tp-AgNPs were generally more effective across various bacterial strains, although their antibacterial activity still fell short of the broad-spectrum inhibition exhibited by chloramphenicol. Gram-negative bacteria were more sensitive to AgNPs than Gram-positive bacteria, likely due to their thinner cell membranes, which allow easier nanoparticle diffusion. In contrast, Gram-positive bacteria possess a thicker peptidoglycan layer that impedes nanoparticle entry. The enhanced permeability in Gram-negative bacteria enables AgNPs to reach the cytoplasm, where they interact with sulfur-containing enzymes and proteins, disrupt cellular functions, and interfere with DNA replication (Durán et al., 2016). The minimum inhibitory concentration (MIC) and minimum bactericidal concentration (MBC) values for Tp-AgNPs, Tm-AgNPs, and Pg-AgNPs against various bacterial strains revealed distinct antibacterial efficacies (Table 3). Tp-AgNPs exhibited the highest potency, particularly against *P. aeruginosa* (MIC: 0.039 mg/mL, MBC: 0.62 mg/mL) and *S. epidermidis* (MIC: 0.15 mg/mL, MBC: 0.31 mg/mL), indicating strong bactericidal activity. For *S. aureus*, Tp-AgNPs showed low MIC values (0.15 mg/mL) but required higher MBC (2.5 mg/mL) to achieve bactericidal effects. Among Gram-negative bacteria, *E. coli* demonstrated moderate sensitivity to Tp-AgNPs (MIC: 0.31 mg/mL, MBC: 0.62 mg/mL), while *S. marcescens* required slightly higher concentrations (MIC: 0.31 mg/mL, MBC: 0.62 mg/mL). Notably, Tm-AgNPs and Pg-AgNPs exhibited reduced efficacy across most strains, requiring higher concentrations to achieve similar inhibitory and bactericidal effects. These results underscored the variability in antibacterial activity depending on the type of nanoparticle and bacterial strain, with Tp-AgNPs emerging as the most effective overall.

AgNPs exert their antibacterial effects through multiple proposed mechanisms. One mechanism involves the attachment of AgNPs to the bacterial cell membrane, leading to structural and functional disruption. This interaction is followed by the invagination of the cell and the release of silver ions, which possess potent antibacterial activity, particularly effective against Gram-negative bacteria due to their thinner cell walls (Singhal et al., 2021). Another pathway suggests that AgNPs anchor to the bacterial cell wall, penetrate the cell, and disrupt internal processes, ultimately leading to cell death. Furthermore, AgNPs can generate free radicals, which exacerbate their bactericidal effects by damaging cellular components such as proteins, lipids, and DNA, and inducing oxidative stress. These combined actions underline the multifaceted antimicrobial potential of AgNPs, making them effective against a broad spectrum of bacterial pathogens (Singhal et al., 2021).

Several studies highlight the synthesis of AgNPs using *P. granatum* peel extract, demonstrating significant antimicrobial efficacy against both Gram-positive and Gram-negative bacteria. AgNPs derived from the aqueous peel extract exhibited notable inhibition zone diameters, with concentrations of 100 µg/mL effectively inhibiting the growth of *E. coli* and *P. aeruginosa*. Remarkably, even at lower concentrations of 25 and 50 µg/mL, the AgNPs inhibited *Klebsiella pneumoniae*, producing inhibition zones of nine mm and 14 mm, respectively. Similarly, against *S. aureus*, inhibition zones of six mm and 14 mm were observed at these concentrations, underlining the potent antimicrobial activity of these AgNPs (Devanesan et al., 2018). Moreover, AgNPs synthesized using *P. granatum* extract demonstrated higher susceptibility in Gram-negative bacteria compared to Gram-positive ones, with MIC values ranging from 16 to 512 µg/mL (Aygün et al., 2022). In a comparative study, the antibacterial activity of silver nanoparticles synthesized with *T. polium* extract and a 96% ethanol extract was tested against *E. coli* K-12 and *S. aureus*. The results revealed that the plant extract significantly enhanced the antibacterial efficacy of the silver nanoparticles, with a 4.2-fold increase in activity against Escherichia coli and a 6.6-fold increase against *S. aureus* (Kazaryan, Rshtuni & Hovhannisyan, 2021). These findings emphasized the enhanced antimicrobial potential of plant extract-mediated AgNPs and their synergistic effects when combined with bioactive plant compounds.

##### Anti-biofilm activity.

A biofilm is a structured community of bacterial cells that adhere to a surface and are embedded within a self-produced matrix of EPS. This matrix consists of exopolysaccharides, particulates, and microbial lysates. Biofilm formation provides bacteria with enhanced resistance to environmental stresses, such as fluctuations in nutrients and oxygen levels, pH changes, and antibiotic treatments (Shahwany, Tawfeeq & Hamed, 2016). This protective mechanism significantly contributes to bacterial survival in extreme environments, both within and outside the host, and is a major factor in the persistence and chronic nature of certain infections (Singhal et al., 2021).

**Figure 7:**
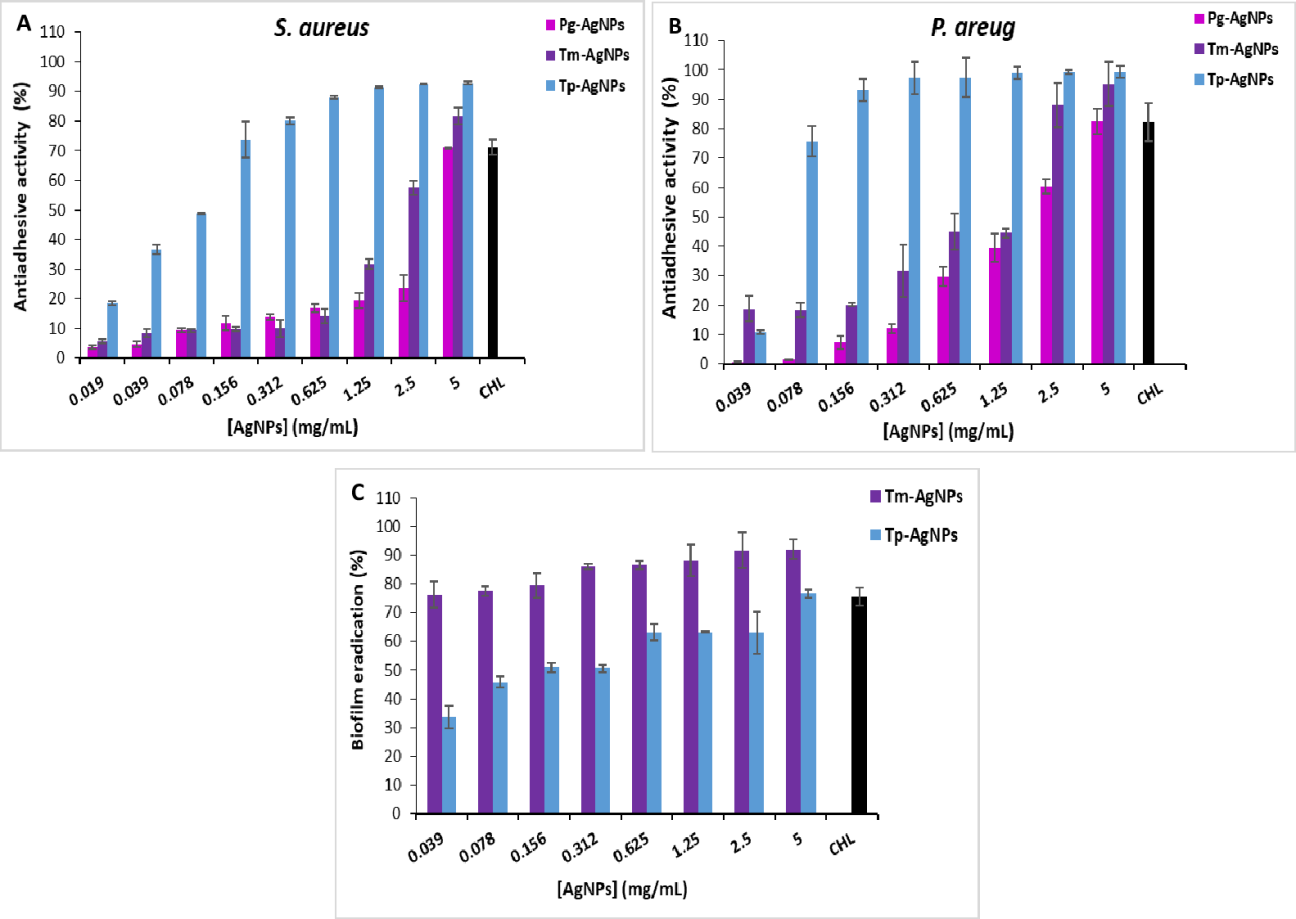
Anti-adhesive effect of AgNPs (Tp-AgNPs, Tm-AgNPs, and Pg-AgNPs) against biofilm of *Staphylococcus aureus* (A) and *Pseudomonas aeruginosa* (B); (C) Eradication effect of Tp-AgNPs, Tm-AgNPs against *Staphylococcus aureus* biofilm. Chloramphenicol (CHL) used as positive control. The results were reported as the mean values along with their corresponding standard deviations.

In this study, *P. aeruginosa* and *S. aureus* strains were exposed to biosynthesized AgNPs, and their ability to form biofilms was evaluated. The results, illustrated in Fig. 7, demonstrate the anti-biofilm activity of three types of nanoparticles: Pg-AgNPs, Tp-AgNPs, and Tm-AgNPs. The effects were assessed separately for *S. aureus* (Fig. 7A) and *P. aeruginosa* (Fig. 7B), highlighting their potential in disrupting biofilm formation and reducing bacterial resilience in challenging environments. Compared to chloramphenicol, which exhibited a 71 ± 2.5% biofilm inhibition at 2.5 mg/mL, Tp-AgNPs demonstrated higher inhibition against *S. aureus*, achieving over 92 ± 0.54% inhibition at concentrations as low as 1.25 mg/mL and maintaining significant effects even at reduced doses (73.64 ± 6.09% at 0.156 mg/mL) (Fig. 7A). Tm-AgNPs showed moderate inhibitory effects, peaking at 81.56 ± 2.83% at five mg/mL, with minimal activity observed at lower concentrations. In contrast, Pg-AgNPs displayed the least inhibitory activity against *S. aureus*, achieving notable inhibition only at five mg/mL (71.06 ± 0.023%) (Fig. 7A). For *P. aeruginosa*, Tp-AgNPs again exhibited the highest biofilm inhibition, reaching over 93 ± 3.86% at 0.156 mg/mL and nearly complete inhibition (99.38 ± 1.97%) at five mg/mL, surpassing chloramphenicol (82.2 ± 6.6%) (Fig. 7B). Tm-AgNPs displayed moderate inhibition, peaking at 95.2 ± 7.51% at five mg/mL but with lower efficacy at minimal concentrations. Pg-AgNPs were the least effective, showing substantial inhibition only at higher concentrations, with a peak of 82.4 ± 4.36% at five mg/mL (Fig. 7B). Overall, Tp-AgNPs demonstrated the most potent anti-biofilm activity against both bacterial strains, followed by Tm-AgNPs, while Pg-AgNPs were the least effective, especially at lower doses.

Limited studies have explored the anti-biofilm potential of AgNPs compared to their antimicrobial effects. Singhal et al. (2021) reported that AgNPs biosynthesized using *P. granatum* leaf extract exhibited greater inhibition of biofilm formation in *P. aeruginosa* compared to *S. aureus* at higher concentrations (100 µg/mL) (Singhal et al., 2021). Bacterial biofilm formation is initiated by the production and secretion of exopolysaccharides, which are crucial for biofilm development. Therefore, inhibiting exopolysaccharides synthesis serves as an effective strategy to restrict biofilm formation, forming the basis for evaluating the anti-biofilm activity of AgNPs (Mohanta et al., 2020).

##### Biofilm eradication.

Mature biofilms are highly resistant to antibacterial agents, making them a critical factor in chronic infections that are difficult to treat with conventional antibiotics. The biofilm eradication effects of AgNPs (Tp-AgNPs and Tm-AgNPs) against *S. aureus* biofilms at various concentrations are shown in Fig. 7C. In this assay, *S. aureus* biofilms were first established in 96-well microtiter plates and subsequently treated with the NPs to evaluate their ability to disrupt mature biofilms. Tm-AgNPs demonstrated significantly greater biofilm eradication capacity across all tested concentrations compared to Tp-AgNPs. At the lowest concentration (0.039 mg/mL), Tm-AgNPs achieved 76.34 ± 1.5% eradication, while Tp-AgNPs achieved only 33.58 ± 3.86% (Fig. 7C). As concentrations increased, the efficacy of both NPs was improved, but Tm-AgNPs consistently outperformed Tp-AgNPs. At 5 mg/mL, Tm-AgNPs reached 91.98 ± 3.29% eradication, surpassing chloramphenicol, the positive control, which showed 75.5 ± 3.24% eradication at the same concentration. Tp-AgNPs, at 5 mg/mL, achieved 76.64 ± 4.69% eradication.

The superior performance of Tm-AgNPs could be attributed to differences in nanoparticle synthesis, size, or composition, which may enhance their interaction with the biofilm matrix. Both Tp-AgNPs and Tm-AgNPs were small enough, in comparison with Pg-AgNPs, to penetrate biofilm structures, disrupting the matrix and diffusing through the extracellular polysaccharides that typically act as barriers to antibacterial agents. Additionally, the eradication of biofilms is likely due to a synergistic effect between the AgNPs and phytochemicals derived from *T. polium* and *T. marum* extracts, further enhancing their biofilm-disrupting properties (Altaf et al., 2021).

These findings were consistent with previous studies. For instance, a study demonstrated that established biofilms of *P. aeruginosa*, *E. coli*, methicillin-resistant *S. aureus*, and *Listeria monocytogenes* were disrupted by 65%, 45%, 64%, and 49%, respectively, using AgNPs (Altaf et al., 2021). Similarly, AgNPs synthesized with *Bothriochloa laguroides* showed up to 80% eradication efficiency against *Yersinia enterocolitica* and *S. aureus* (Toranzo et al., 2022). These results underscored the potential of AgNPs as effective agents for biofilm disruption and highlighted their promising role in managing persistent infections.

#### Anti-tumoral effect

Breast carcinoma remains a major health concern with rising incidence rates, and current treatments are limited to conventional radiotherapy and chemotherapy. These therapies often lack selectivity, affecting both cancerous and healthy cells, which underscores the need for innovative treatment options (Jang et al., 2016). NPs, with their unique properties, offer a novel approach to tumor detection, prevention, and treatment by targeting cancer cells through blood vessels, penetrating tumor regions, and reaching the target cells (Devanesan et al., 2018; Jannathul Firdhouse & Lalitha, 2015).

**Figure 8:**
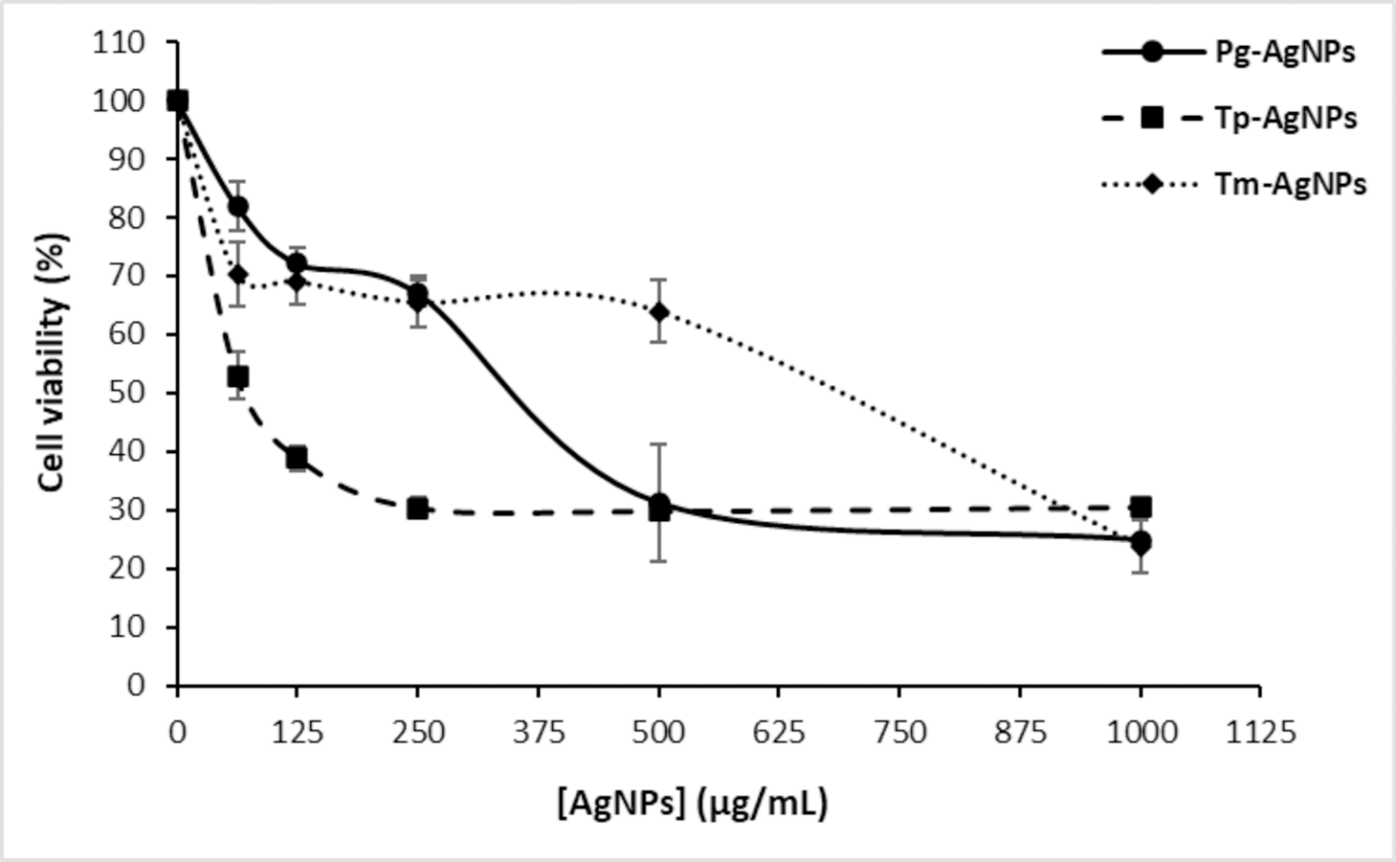
Anti-tumoral effect of AgNPs (Tp-AgNPs, Tm-AgNPs, and Pg-AgNPs) against human breast cancer cell MCF-7. The results are reported as the mean values along with their corresponding standard deviations.

The current data demonstrated the dose-dependent cytotoxic effects of Tp-AgNPs, Pg-AgNPs, and Tm-AgNPs on MCF-7 breast cancer cell lines (Fig. 8). At the lowest tested concentration of 62.5 µg/mL, cell viability remained relatively high for Pg-AgNPs and Tm-AgNPs, with values of 81.94 ± 4.34% and 70.25 ± 5.34%, respectively. In contrast, Tp-AgNPs exhibited significantly higher cytotoxicity, reducing cell viability to 52.96 ± 4.05%. As the concentration increased, all three NPs showed a dose-dependent reduction in cell viability. At 250 µg/mL, Tp-AgNPs maintained the most potent cytotoxicity, reducing viability to 30.34 ± 1.73%, while Pg-AgNPs and Tm-AgNPs displayed slightly higher viabilities of 67.03 ± 2.2% and 65.50 ± 4.33%, respectively. At the highest concentration of 1,000 µg/mL, all NPs demonstrated substantial cytotoxic effects, with Tm-AgNPs achieving the lowest cell viability at 23.79 ± 4.84%. Pg-AgNPs and Tp-AgNPs showed comparable effects at this concentration, with viabilities of 24.82 ± 0.58% and 30.50 ± 0.45%, respectively.

These results indicated that while all three AgNPs exhibit cytotoxic effects against MCF-7 cells, Tp-AgNPs are more effective at lower concentrations, with an IC_50_ value of 45.7 ± 2.93 µg/mL. Conversely, Tm-AgNPs demonstrated enhanced cytotoxicity at higher concentrations. The observed dose-dependent cytotoxicity underscored the potential of these AgNPs as anticancer agents and highlighted the need for further investigation into their mechanisms of action. These findings reinforce the promising role of AgNPs as dose-responsive therapeutic agents against MCF-7 cell lines.

According to previous studies, the anti-tumor effects of silver nanoparticles are linked to several mechanisms, including damage to nucleic acids and organelles, induction of oxidative stress, initiation of apoptosis, and disruption of mitochondrial function (Ong et al., 2013; Amini et al., 2021). AgNPs can enter cancer cells through endocytosis and accumulate in the perinuclear area and endo-lysosomal compartments. They can also reach the mitochondria, where they interfere with the respiratory chain, leading to the production of ROS. These ROS can damage proteins, lipids, and nucleic acids, as well as compromise cell membranes, ultimately activating apoptotic pathways and resulting in cell death (Yeşilot & Aydın Acar, 2019). Current findings align with several studies investigating AgNPs synthesized using similar plant extracts. For instance, AgNPs derived from the aqueous leaf extract of *P. granatum* effectively inhibited HeLa cell proliferation, with an IC50 value of 100 µg/mL (Sarkar & Kotteeswaran, 2018). Similarly, *P. granatum* peel extract AgNPs demonstrated significant cytotoxicity against the RKO colon cancer cell line, reducing cell viability to 61% at concentrations above 12.5 µg/mL (Devanesan et al., 2018). Furthermore, AgNPs synthesized using *T. polium* leaf extract exhibited potent anticancer effects against the MNK45 human gastric cancer cell line, achieving an IC_50_ value of 68.2 µg/mL (Hashemi, Tasharrofi & Saber, 2020). Amini et al. (2021), 30 also reported that *T. polium*-based AgNPs significantly reduced NALM-6 leukemia cell viability at concentrations ≥ 50 µg/mL by inducing apoptosis, while sparing normal peripheral blood mononuclear cells.

Moreover, a notable property of NPs is that their biological impact varies considerably based on their size, which, in turn, plays a critical role in determining their efficiency of cellular uptake, from the intracellular membrane to the nuclear membrane (Kim et al., 2012). It has been documented that the cytotoxic effects of various metallic nanoparticles, such as gold and silver NPs, on human cancer cell lines are size-dependent (Kim et al., 2012; Yuan et al., 2010). Therefore, the cytotoxic effects observed for the three AgNPs against MCF-7 cells could be attributed to their relatively larger size in comparison to that of the cells. Larger NPs have a higher bioactive compound-to-NP ratio, which facilitates the reorganization of these compounds on their surface to optimize their loading into cells (Jiang et al., 2008).

All together, these studies corroborate the cytotoxic potential of Ag-NPs derived from plant extracts on cancer cell line, highlighting their selective action and promising role as anticancer agents.

## Conclusion

XRD analysis confirmed that the biosynthesized Ag-NPs possessed a face-centered cubic crystal structure, with crystallite sizes smaller than the hydrodynamic diameters observed in DLS, likely due to additional surface layers. DLS analysis revealed monodisperse distributions for Tp-AgNPs and Tm-AgNPs, while Pg-AgNPs exhibited fewer uniform distributions. Autocorrelation decay patterns indicated smaller, faster particles for Tp-AgNPs and Tm-AgNPs, compared to larger, slower particles for Pg-AgNPs. In biological assays, Tp-AgNPs demonstrated the strongest antibacterial activity, particularly against Gram-negative bacteria, although their efficacy was lower than that of chloramphenicol. In biofilm inhibition studies, Tp-AgNPs were the most effective, especially against *S. aureus* and *P. aeruginosa*, followed by Tm-AgNPs. Pg-AgNPs were the least effective, particularly at lower concentrations. Cytotoxicity assays on MCF-7 breast cancer cells revealed that Tm-AgNPs exhibited the highest anticancer activity, followed by Tp-AgNPs. Collectively, Tp-AgNPs and Tm-AgNPs demonstrated significant antimicrobial, anti-biofilm, and anticancer properties, highlighting their potential for biomedical applications. However, further research is needed to evaluate their clinical safety and therapeutic efficacy.

##  Supplemental Information

10.7717/peerj.19608/supp-1 Supplemental Information 1 Raw data

## Additional Information and Declarations

### Data Availability

The following information was supplied regarding data availability: The raw measurements are available in the Supplementary File.
